# Effects of COVID-19 pandemics on urinary metabolites in kidney stone patients: our kidney stone prevention clinic experience

**DOI:** 10.1186/s12199-021-01037-w

**Published:** 2021-12-02

**Authors:** Sanaz Tavasoli, Nasrin Borumandnia, Abbas Basiri, Maryam Taheri

**Affiliations:** Urology and Nephrology Research Center, Shahid Beheshti University of Medical Sciences, Tehran, IR Iran

**Keywords:** COVID-19, Diet, Iran, Kidney stone, Prevention, 24-h urine

## Abstract

**Background:**

The dietary habits and lifestyle changes during the COVID-19 pandemic could affect the urinary risk factors in kidney stone formers. In this study, we investigated the effects of the COVID-19 pandemic on 24-h urine metabolites, as a surrogate for dietary intake, in patients with kidney stones, in Tehran, Iran.

**Methods:**

We evaluated the medical records of all patients with urolithiasis who visited in our stone prevention clinic from the beginning of COVID-19 in Iran to 1 year later (Feb 2020–Feb 2021) and compared it with the patients’ medical records in the same period a year before COVID-19 (Feb 2019–Feb 2020).

**Results:**

The results of our stone prevention clinic showed a decrease in the number of visits during COVID-19. Twenty-four-hour urine urea, sodium, and potassium were significantly lower, and 24-h urine magnesium was significantly higher during COVID-19. Higher 24-h urine oxalate was only shown in patients with the first-time visit, whereas lower 24-h urine uric acid and citrate were only shown in patients with the follow-up visits.

**Conclusions:**

COVID-19 pandemics may change some of the dietary habits of the patients, including lower salt, protein, and fruit and vegetable intake. Although economic issues, restricted access, or sanitation issues may be the reason for the undesirable dietary changes, the importance of a quality diet should be discussed with all patients, as possible. Since the number of patients visited in the stone clinic was lower during COVID-19, virtual visits could be an excellent alternative to motivate patients with kidney stones.

## Background

It is now clear that the coronavirus disease 2019 (COVID-19) pandemic is a challenging and long-standing healthcare problem rather than a temporary condition [1]. Iran is one of the countries severely affected by the COVID-19 pandemic [2]. The COVID-19 pandemic influences different aspects of human beings’ lifestyle and health. Most of the healthcare resources are focused on COVID-19 globally [3]. The COVID-19 outbreak could increase stress among all population groups, with certain groups, such as patients with chronic diseases, at higher risk [2]. Social limitations have adverse effects on mental health, physical activity, and dietary habits [4]. All of these changes could impair the care of patients with chronic diseases [3].

Kidney stone disease has a high prevalence, incidence, and recurrence rate in the recent decade [5, 6]. Because the disease and its recurrence cause a high burden [7], prevention is of utmost importance in this condition [8]. The main target of preventive recommendations is to modify dietary habits and reduce lifestyle risk factors [9]. In patients with a high risk of recurrence, an extensive metabolic workup is recommended to identify stone risk factors and initiate a specific diet and medical treatments for each patient. Generally, kidney stone patients are recommended to have high fluid, high fruit and vegetable, restricted sodium, and adequate calcium intake. Besides, all patients should be encouraged to have adequate physical activity and keep a normal weight [9].

Such as other chronic diseases, the treatment and prevention of kidney stones have markedly changed during the COVID-19 pandemic [1]. The European Association of Urology (EAU) considered metabolic evaluation a low priority action during COVID-19 [10]. The panel recommended that stone analysis should be performed in first-time stone formers using a valid procedure, and extensive metabolic evaluation should be postponed. However, it is noteworthy that this recommendation should not diminish the importance of kidney stone prevention since low compliance with stone prevention recommendations might lead to stone recurrence, renal colic, urologic emergencies, and kidney injuries. Furthermore, the dietary and lifestyle changes caused by pandemics may independently be the risk factors for kidney stone formation. Therefore, we must balance the risk of kidney injures or emergent urologic interventions due to postponing stone clinic visits against the risk of COVID-19 exposure because of stone clinic appointments.

Some of the dietary habit changes during the COVID-19 outbreak may be risk factors for kidney stone formation. The stress-caused overeating and carbohydrate craving [11] increase the risk of developing obesity. Moreover, studies showed a decreased intake of vegetables and some types of fruit [12] and increased consumption of processed foods, high in fat, sugar, and salt [11–13]. All of these changes could increase the risk of stone recurrence. However, data regarding the dietary changes during pandemics is limited and controversial.

According to the studies, some of the 24-h urine (24-U) metabolites reflect patients' diet and can be used as a surrogate for their dietary intake. These metabolites include urine volume (primary determinant of fluid intake), urea (a marker of total protein intake), uric acid (could be used as a marker of animal protein), magnesium (reflecting the intake of whole grains, legumes, and nuts), and potassium (reflecting the intake of vegetables and fruits) [14–17].

In the current study, we compared the 24-U metabolites of patients during and before COVID-19 pandemics to investigate the dietary changes during the COVID-19 era in our stone prevention clinic in Tehran, Iran.

## Methods

We reviewed the medical records of all patients visited in our stone prevention clinic from the beginning of COVID-19 in Iran to one year later, i.e., 20 Feb 2020 to 19 Feb 2021 (during the COVID-19 group). As a control group, patients’ medical records from 20 Feb 2019 to 19 Feb 2020 were also reviewed (Pre COVID-19 group).

Our stone prevention clinic is a tertiary center located in Shahid Labbafinejad Hospital, Tehran, Iran. According to international stone prevention guidelines [9, 14], all patients referred to our clinic have a screening evaluation at their first visit consisting of a detailed medical history, stone analysis (if available), serum chemistries, and two consecutive 24-U analyses. After that, all patients will have a targeted diet and medical treatment, according to guidelines. All patients will have a follow-up visit eight to twelve weeks after initiating treatment to evaluate compliance to diet and response to treatments. Later follow-up visits will be scheduled according to each patient's stone activity and response to medical therapy. During COVID-19 pandemics, we changed our management strategy according to an expert panel consensus. First, in the initial screening evaluation, we asked patients to perform only one 24-U analysis. Second, the stone prevention clinic was closed during each COVID-19 peak, and we only had online visits.

The number of visited patients during each period, age, gender, the stone clinic visit status (first-time visit vs. follow-up visit), and 24-U metabolites were collected from patients’ records. 24-U metabolites (including creatinine, uric acid, calcium, magnesium, phosphate, sodium, potassium, citrate, and oxalate) were also extracted from patients’ records and compared between groups. Only the results of adult patients (age >18 years) and 24-U results with a proper collection [18] were included in the analyses. As mentioned earlier, we used 24-U analyses as a substitute for patients’ dietary intake. Given that dietary recommendations by the stone clinic could change patients’ knowledge and behavior, we divided patients into two groups according to the stone clinic visit’s status (first-time and follow-up groups) and compared the 24-U metabolites during and before COVID-19 pandemics in each group.

The normality of data was analyzed using Shapiro–Wilk test. The quantitative variables were compared between groups using an independent sample *T* test (or Mann-Whitney *U* test for skewed data). The association between quantitative variables was tested using the chi-square test. Statistical analyses were performed with the IBM SPSS Statistics software, version 24 (IBM Corp., NY, USA). The *p* value < 0.05 was set as statistical significance.

## Results

The number of visited patients during COVID-19 and pre COVID-19 was 672 and 1625, respectively. There was no significant difference between groups regarding age and sex (Table 1). The number of first-time visits was significantly reduced during the COVID-19 period (*p* = 0.005).

**Table 1:** Demographic variables of kidney stone formers during and before COVID-19

Variable	Before COVID-19	During COVID-19	***p*** value
Number of visits	1625	672	
First-time visit	434 (26.7%)	142 (21.1%)	0.005** ^a^
Follow-up visit	1191 (73.3%)	530 (78.9%)	
Age, Mean (SD)	48.68 (14.02)	48.29 (14.36)	0.707 ^b^
First-time visit	47.61 (14.07)	45.15 (14.35)	0.073 ^b^
Follow-up visit	50.37 (13.82)	51.83 (13.61)	0.324 ^b^
Male patients, Number (%)	1033 (71.4%)	413 (28.6%)	0.341 ^a^
First-time visit	265 (73.6%)	95 (26.4%)	0.212 ^a^
Follow-up visit	768 (70.7%)	318 (29.3%)	0.075 ^a^

Bold values emphasize statistical significance

^a^*p* value stands for chi-square test

^b^*p* value stands for an independent sample *T* test

**p value <0.01

Tables 2 and 3 compare 24-U metabolites between groups in first-time and follow-up visits, respectively. In patients with the first-time visit, 24-U urea, sodium, and potassium were significantly lower during COVID-19 (*p*=0.012, *p*=0.005, and *p*=0.002, respectively). Conversely, 24-U magnesium and oxalate were higher in the first time visited patients during COVID-19 (*p*<0.001 and *p*=0.001, respectively) (Table 2). 24-U uric acid and citrate were lower during COVID-19; however, the differences were not significant (*p*=0.257 and *p*=0.407, respectively) (Table 2).

**Table 2:** Twenty-four-hour urine metabolites of kidney stone formers having first-time stone clinic visit during and before COVID-19

Variable	Before COVID-19 (***n***=336)	During COVID-19 (***n***=113)	***p*** value^**a**^
24-U volume	1725 (756)	1795 (700)	0.386
24-U creatinine	1.17 (0.36)	1.26 (0.35)	0.055
24-U urea	27.05 (8.98)	24.54 (8.77)	0.012*
24-U sodium	156.43 (67.98)	135.22 (64.44)	0.005**
24-U potassium	52.04 (20.08)	43.10 (26.85)	0.002**
24-U uric acid	441.27 (173.26)	414.43 (229.65)	0.257
24-U magnesium	72.89 (29.07)	97.38 (31.84)	<0.001***
24-U calcium	185.70 (94.84)	196.63 (112.22)	0.358
24-U oxalate	37.48 (18.23)	44.07 (18.32)	0.001***
24-U citrate	742.4 (347.0)	710.1 (370.4)	0.407

Bold values emphasize statistical significance

*24-U* 24-h urine

^a^All *p* values stand for an independent sample *T* test

*p value <0.05

**p value <0.01

***p value <0.001

**Table 3:** Twenty-four-hour urine metabolites of kidney stone formers having follow-up stone clinic visit during and before COVID-19

Variable	Before COVID-19 (***n***=1088)	During COVID-19 (***n***=469)	***p*** value^**a**^
24-U volume	2226 (790)	2156 (771)	0.108
24-U creatinine	1.19 (0.36)	1.15 (0.34)	0.110
24-U urea	30.77 (9.84)	27.06 (10.10)	<0.001***
24-U sodium	161.74 (71.30)	151.45 (68.81)	0.009**
24-U potassium	59.28 (24.64)	53.80 (27.18)	<0.001***
24-U uric acid	449.40 (169.36)	415.50 (173.65)	<0.001***
24-U magnesium	80.51 (28.71)	101.36 (37.43)	<0.001***
24-U calcium	220.31 (100.09)	213.89 (100.42)	0.249
24-U oxalate	46.05 (19.35)	45.07 (19.39)	0.359
24-U citrate	821.7 (344.0)	762.4 (370.2)	0.003**

Bold values emphasize statistical significance

*24-U* 24-h urine

^a^All *p* values stand for an independent sample *T* test

**p value <0.01

***p value <0.001

Table 3 shows the results of patients having follow-up visits during COVID-19. As shown, 24-U urea, sodium, potassium, uric acid, and citrate were significantly lower during COVID-19 (*p*<0.001, *p*=0.009, *p*<0.001, *p*<0.001, and *p*=0.003, respectively). Similar to patients having the first-time visit, 24-U magnesium was higher during COVID-19 (*p*<0.001) (Table 3).

Neither the first-time visit group nor the follow-up visit group had any significant 24-U volume and calcium changes during COVID-19.

## Discussion

Our results showed that fewer patients were visited during the COVID-19 pandemic. This decline was significantly greater in first-time visit patients. It may be both because of social limitations and the cancelation of appointments [19]. Decrease in chronic disease care is one of the concerns during COVID-19 [3, 19]. Since follow-up visits not only assess the efficacy of treatment but also increase patient compliance to the dietary recommendation and medical treatment [20], fewer visits could lead to higher stone recurrence rates and more required urologic interventions. Teleconsultation and online follow-up visits could be alternative methods to meet patients’ needs and increase their compliance [21].

In this study, both first-time and follow-up groups showed lower 24-U potassium during COVID-19. Besides, follow-up patients had lower 24-U citrate during COVID-19. Both 24-U potassium and citrate are shown to be correlated with dietary fruit and vegetable intake [22]. Therefore, lower potassium and citrate may be due to lower vegetable and fruit intake during COVID-19. Other studies also reported decreased vegetable and fruit intake during COVID-19 [12]. A decline in fruit and vegetable intake could increase kidney stone recurrence [22]. Restricted access to fresh food because of lockdown, economic problems, or sanitation issues may be the reason for decreased vegetable and fruit intake [12]. However, the importance of a quality diet should be discussed with patients, and they should be encouraged to have a high intake of vegetables and fruits.

Regarding 24-U urea and uric acid during COVID-19, both groups had lower 24-U urea, and the follow-up group showed lower uric acid than the pre-COVID-19 group. Low 24-U urea and uric acid may reflect lower total protein and animal protein intake, resulting from pandemics’ economic impact. The low dietary content of non-dairy animal proteins is recommended for kidney stone prevention. However, since dairy products are also good dietary protein sources, their intake also may be reduced. Dairy products are good sources of dietary calcium and are shown to reduce the risk of kidney stone formation [23].

In our study, both groups showed higher 24-U magnesium, which may be due to a higher intake of whole grains, legumes, and nuts [16]. Magnesium is an inhibitor of crystallization, and higher 24-U magnesium is protective against kidney stone formation. However, some dietary magnesium sources such as nuts are high in oxalate, and their high intake could cause hyperoxaluria [24] and be the reason for higher 24-U oxalate during COVID-19 in first-time visit patients.

Some recent studies reported that consumption of salty snacks and processed foods might be increased during pandemics [11–13]. Unlike these findings, our results showed that 24-U sodium was lower during COVID-19 in both groups. Low 24-U sodium could decrease kidney stone formation and recurrence risk, and all patients should be recommended to limit their sodium intake [9].

As mentioned before, the EAU guideline recommended postponing the metabolic evaluation in kidney stone patients [10]. However, previous studies showed that targeted nutrition therapy based on patients’ metabolic abnormalities was more successful than general dietary recommendations in preventing stone recurrence [20]. Using telemedicine and virtual stone clinics could provide good care in the era of the pandemic situation [25]. It is shown that patients had high compliance to virtual visits [19]. The remaining question is the effect of postponing urine metabolic evaluation and using empiric therapy on the stone recurrence rate in kidney stone patients, which needs more studies.

An important point that should be mentioned is that the patients who attended their appointments had higher compliance to stone prevention routines. We do not have any information about the people who missed their appointments, which may have lower compliance to stone prevention recommendations. Using telemedicine and virtual stone clinics could provide care to this group of patients [25].

To the best of our knowledge, this is the first study that evaluated the effect of COVID-19 pandemics on the 24-U metabolites in kidney stone patients. The main limitation of the study was that we did not have any data regarding patients who missed their clinic appointments. Moreover, using information from food frequency questionnaires and 24-h dietary recalls together with 24-h urine metabolites could better reflect the dietary intake of patients during COVID-19.

## Conclusions

Our results showed that COVID-19 pandemics changed some of the dietary habits of kidney stone patients. Lower 24-U potassium and citrate, which reflects lower fruit and vegetable intake, and higher 24-U oxalate could increase stone recurrence. This finding is a concern, particularly in complicated patients with recurrent kidney stones. Therefore, the medical systems should consider the concern and have proper educational material for patients with chronic conditions, such as recommending a quality diet in kidney stone formers. Since the number of patients visited in our stone prevention clinic was lower during COVID-19, virtual visits could be an excellent alternative to motivate patients to follow stone prevention recommendations and medical treatment.

## Data Availability

The datasets used and/or analyzed during the current study are available from the corresponding author on reasonable request.

## Abbreviations

24-U: 24-h urine
COVID-19: Coronavirus disease 2019
EAU: European Association of Urology
